# Synthesis of β-Maltooligosaccharides of Glycitein and Daidzein and their Anti-Oxidant and Anti-Allergic Activities

**DOI:** 10.3390/molecules15085153

**Published:** 2010-07-29

**Authors:** Kei Shimoda, Hiroki Hamada

**Affiliations:** 1 Department of Chemistry, Faculty of Medicine, Oita University, 1-1 Hasama-machi, Oita 879-5593, Japan; 2 Department of Life Science, Okayama University of Science, 1-1 Ridai-cho, Kita-ku, Okayama 700-0005, Japan

**Keywords:** glycitein, daidzein, β-maltooligosaccharides, anti-allergic activity, anti-oxidant activity

## Abstract

The production of β-maltooligosaccharides of glycitein and daidzein using *Lactobacillus delbrueckii* and cyclodextrin glucanotransferase (CGTase) as biocatalysts was investigated. The cells of *L. delbrueckii* glucosylated glycitein and daidzein to give their corresponding 4'- and 7-*O*-β-glucosides. The β-glucosides of glycitein and daidzein were converted into the corresponding β-maltooligosides by CGTase. The 7-*O*-β-glucosides of glycitein and daidzein and 7-*O*-β-maltoside of glycitein showed inhibitory effects on IgE antibody production. On the other hand, β-glucosides of glycitein and daidzein exerted 2,2-diphenyl-1-picrylhydrazyl (DPPH) free-radical scavenging activity and supeoxide-radical scavenging activity.

## 1. Introduction

Glycitein and daidzein are important and bioactive isoflavones isolated from soybeans whose pharmacological properties such as anticancer, anti-inflammatory, neuroprotective, anticarcinogenic effects, and protective effects against bone loss, hormone-dependent and -independent cancers, cardiovascular diseases, and autoimmune diseases and have been widely studied [1,2,3,4,5,6,7,8,9,10,11]. Despite these pharmacological activities, their use as medicines and functional food-ingredients is limited, because they are scarcely soluble in aqueous solution and poorly absorbed through oral administration.

Glycosylation is an important method for the conversion of water-insoluble and unstable organic compounds into the corresponding water-soluble and chemically stable derivatives. Mizukami *et al.* reported that glucosyl conjugation was far more effective than cyclodextrin complexation at enhancing the water solubility of hydrophobic compounds such as curcumin [12]. Recently, absorption efficiency of a lipophilic flavonoid, *i.e.*, quercetin, has been reported to be much improved, when converted into its glycoconjugates [13,14]. Glycosylations of glycitein and daidzein are of importance from the viewpoint of pharmacological development of soy isoflavones. We report here the synthesis of β-maltooligosaccharides of glycitein and daidzein by sequential glycosylation with *Lactobacillus delbrueckii* and cyclodextrin glucanotransferase (CGTase). We also report their inhibitory activity for IgE antibody formation, 2,2-diphenyl-1-picrylhydrazyl (DPPH) radical scavenging activity, and supeoxide-radical scavenging activity.

## 2. Results and Discussion

### 2.1. Synthesis of β-maltooligosaccharides of glycitein and daidzein

**Figure 1:**
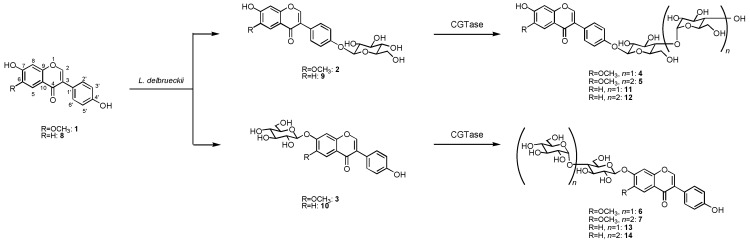
Synthesis of β-maltooligosaccharides of glycitein (**1**) and daidzein (**8**) by glycosylation with *L. delbrueckii* and CGTase.

The biotransformation of glycitein (**1**) was investigated using the cultured cells of *L. delbrueckii*. When cells of *L. delbrueckii *were incubated with glycitein (**1**) in the presence of glucose for 5 days, two products, **2 **and **3**, were isolated from the *n*-butanol extracts of cell cultures by preparative HPLC (**2**, 5% yield; **3**, 7%). The chemical structures of **2 **and **3 **were determined by spectroscopic methods such as HRFABMS, ^1^H- and ^13^C-NMR as glycitein 4'-*O*-β-D-glucoside (**2**) [15] and glycitein 7-*O*-β-D-glucoside (**3**) [15]. Next, the glucoside product **2** was further glycosylated by cyclodextrin glucanotransferase (CGTase) to give two new compounds **4 **and **5** in 45 and 39% yield, respectively. The structures of products **4 **and **5** were identified as glycitein 4'-*O*-β-maltoside and glycitein 4'-*O*-β-maltotrioside, which have not been reported before, by HRFABMS, ^1^H- and ^13^C-NMR, H-H COSY, C-H COSY, and HMBC spectra. Also, the glucoside **3** was converted by CGTase to give glycitein 7-*O*-β-maltoside (**6**) and glycitein 7-*O*-β-maltotrioside (**7**), which were two new compounds, in 41 and 33% yield, as shown in Figure 1.

The HRFABMS spectra of **4 **and **6** included pseudomolecular ion [M+Na]^+^ peaks at 631.1640 for **4** and 631.1641 for **6 **(calculated for C_2__8_H_32_O_1__5_Na, 631.1639), indicating that each product consisted of one substrate and two hexoses. The sugar components in these products were determined to be glucose on the basis of their chemical shifts of the carbon signals. The ^1^H-NMR spectra showed two proton signals at *δ* 5.05 (1H, *d*, *J *= 3.2 Hz) and 5.11 (1H, *d*, *J *= 7.6 Hz) for **4**, and *δ* 5.07 (1H, *d*, *J *= 3.2 Hz) and 5.19 (1H, *d*, *J *= 7.6 Hz) for **6**, indicating that the glucoside linkage in these compounds had each of α- and β-orientations. The HMBC spectra of **4 **and **6** included correlations between the proton signal at *δ* 5.05 (H-1''') and the carbon signal at *δ* 80.5 (C-4'') and between the proton signal at *δ* 5.11 (H-1'') and the carbon signal at *δ* 157.3 (C-4') for **4**, and between the proton signal at *δ* 5.07 (H-1''') and the carbon signal at *δ* 79.9 (C-4'') and between the proton signal at *δ* 5.19 (H-1'') and the carbon signal at *δ* 152.5 (C-7) for **6**. These data indicated that **4 **and **6** were β-maltosyl analogues of **1**, the sugar moiety of which was attached at their 4'- (**4**) and 7-positions (**6**). Thus, products **4 **and **6** were identified as glycitein 4'-*O*-β-maltoside (**4**) and glycitein 7-*O*-β-maltoside (**6**), respectively.

The HRFABMS spectrum of **5 **and **7** with pseudomolecular ion [M+Na]^+^ peak [*m*/*z* 793.2170 (**5**) and 793.2177 (**7**)] established a molecular formula of C_34_H_42_O_20_ (calcd. 793.2167 for C_34_H_42_O_20_Na), indicating that each product consisted of one substrate and three hexoses. The sugar components in these products were identified as glucose on the basis of the chemical shifts of the carbon signals. The ^1^H-NMR spectra showed three proton signals at *δ* 5.05 (1H, *d*, *J = *3.2 Hz), 5.07 (1H, *d*, *J = *3.0 Hz), and 5.12 (1H, *d*, *J = *7.6 Hz) for **5**, and *δ* 5.07 (1H, *d*, *J = *3.2 Hz), 5.09 (1H, *d*, *J = *3.2 Hz), and 5.19 (1H, *d*, *J = *7.6 Hz) for **7**, indicating that the glucoside linkage in these compounds had two α-orientations and one β-orientation. Correlations were observed in the HMBC spectra of **5 **and **7** between the proton signal at *δ* 5.05 (H-1'''') and the carbon signal at *δ* 78.9 (C-4'''), between the proton signal at *δ* 5.07 (H-1''') and the carbon signal at *δ* 78.8 (C-4''), and between the proton signal at *δ* 5.12 (H-1'') and the carbon signal at *δ* 157.6 (C-4') for **5**, and between the proton signal at *δ* 5.07 (H-1'''') and the carbon signal at *δ* 79.9 (C-4'''), between the proton signal at *δ* 5.09 (H-1''') and the carbon signal at *δ* 79.7 (C-4''), and between the proton signal at *δ* 5.19 (H-1'') and the carbon signal at *δ* 152.5 (C-7) for **7**. These data indicated that **5 **and **7** were β-maltotriosyl analogues of **1**, the sugar moiety of which was attached at their 4'- (**5**) and 7-positions (**7**). Thus, the structures of products **5 **and **7** were determined to be glycitein 4'-*O*-β-maltotrioside (**5**) and glycitein 7-*O*-β-maltotrioside (**6**), respectively.

On the other hand, incubation of daidzein (**8**) with *L. delbrueckii *cells in the presence of glucose for 5 days gave daidzein 4'-*O*-β-D-glucoside (**9**, 5%) [16] and daidzein 7-*O*-β-D-glucoside (**10**, 5%) [16]. The glucoside **9** was converted by CGTase to give daidzein 4'-*O*-β-maltoside (**11**) [16] and daidzein 4'-*O*-β-maltotrioside (**12**) [16] in 46 and 35% yield. Compound **10** was glycosylated to daidzein 7-*O*-β-maltoside (**13**) [16] and daidzein 7-*O*-β-maltotrioside (**14**) [16] in 39 and 30% yield by CGTase (Figure 1).

### 2.2. Anti-allergic activity of β-glycosides of glycitein and daidzein

The effects of glycitein β-glycosides **2**-**7 **on IgE antibody formation were investigated by an *in vivo* bioassay using 7S-globulin from soybean as an antigen [17]. The average rat plasma IgE level after treatment of 7S-globulin with or without test compounds was examined. As shown in Table 1, glycitein 7-*O*-β-D-glucoside (**3**) and glycitein 7-*O*-β-maltoside (**6**) showed inhibitory action on IgE antibody generation. On the other hand, glycitein 4'-*O*-β-D-glucoside (**2**), glycitein 4'-*O*-β-maltoside (**4**), glycitein 4'-*O*-β-maltotrioside (**5**), and glycitein 7-*O*-β-maltotrioside (**7**) did not exhibit the inhibitory action on IgE antibody formation. Next, the effects of daidzein β-glycosides **9**-**14** on IgE antibody generation were investigated using the same bioassay systems described above. Only daidzein 7-*O*-β-D-glucoside (**10**) showed inhibitory activity for IgE antibody formation. The 4'-*O*-β-D-glucoside (**9**), 4'-*O*-β-maltoside (**11**), 4'-*O*-β-maltotrioside (**12**), 7-*O*-β-maltoside (**13**), and 7-*O*-β-maltotrioside (**14**) of daidzein did not inhibit IgE antibody generation (Table 1).

**Table 1:** Suppressive action of β-glycosides of glycitein and daidzein **2**-**7 **and **9**-**14** on IgE antibody formation.

Compound	IgE level^a^
None	392.8 ± 120.7
**2**	382.2 ± 100.5
**3**	176.3 ± 87.0*
**4**	407.7 ± 126.2
**5**	453.0 ± 186.9
**6**	251.1 ±68.8
**7**	356.2 ± 188.8
**9**	397.5 ± 150.7
**10**	164.1 ± 78.5*
**11**	410.6 ± 188.0
**12**	435.7 ± 181.4
**13**	389.9 ± 145.5
**14**	400.8 ± 130.8
Hydrocortisone	341.0 ± 122.5

^a ^The results were expressed as average of plasma IgE level of 7 rats administered a total of 10 mg/kg of each test compound. Data are presented as mean ± SE. An asterisk indicates significant differences from controls (*P < 0.05)

It has been reported that tocopheryl β-glycosides showed inhibitory effects on IgE antibody formation [18,19]. Recently, we reported that 7-*O*-β-glycosides of genistein and quercetin showed anti-allergic activities, whereas the β-glycosides whose sugar is attached at other phenolic hydroxyl groups, exhibited no anti-allergic actions [20]. These findings suggested that the C-7 β-glucoside and β-maltoside of glycitein and/or daidzein did not attenuate the anti-allergic activity, and that phenolic hydroxyl groups at the 4'-position might be necessary for glycosides of glycitein and daidzein to act as anti-allergic species. Studies on the anti-allergic mechanism(s) of the β-glycosides of glycitein and daidzein synthesized here are now in progress.

### 2.3. Anti-oxidant activity of β-glycosides of glycitein and daidzein

The antioxidative activities of glycitein β-glycosides **2**-**7 **and daidzein β-glycosides **9**-**14** were determined by an *in vitro* bioassay of their DPPH radical scavenging activity. The antioxidant activities were expressed as IC_50_ values and are summarized in Table 2. Glycitein 4'-*O*-β-D-glucoside (**2**), glycitein 7-*O*-β-D-glucoside (**3**), daidzein 4'-*O*-β-D-glucoside (**9**), daidzein 7-*O*-β-D-glucoside (**10**) showed DPPH free-radical scavenging activity, whereas the β-maltosides and β-maltotriosides of glycitein and daidzein **4**-**7** and **11**-**14** had no antioxidant activity. The results obtained here suggested that monoglucosides of glycitein and daidzein might be useful free-radical scavenging antioxidants with high aqueous-solubility.

The superoxide-radical scavenging activity of glycitein β-glycosides **2**-**7 **and daidzein β-glycosides **9**-**14** were expressed as IC_50_ values, summarized in Table 2. Glycitein 4'-*O*-β-D-glucoside (**2**), glycitein 7-*O*-β-D-glucoside (**3**), and daidzein 4'-*O*-β-D-glucoside (**9**) showed superoxide-radical scavenging activity. The results obtained here suggested that monoglucosides of glycitein and daidzein could be potential superoxide-radical scavenging antioxidants.

**Table 2:** Antioxidant activities of glycitein β-glycosides **2**-**7 **and daidzein β-glycosides **9**-**14**.

Compound	IC_50_ (μM)	
	DPPH free-radical scavenging	Superoxide-radical scavenging
**2**	55	770
**3**	51	708
**4**	140	>1,000
**5**	>200	>1,000
**6**	>200	>1,000
**7**	>200	>1,000
**9**	76	829
**10**	45	767
**11**	155	>1,000
**12**	>200	>1,000
**13**	>200	>1,000
**14**	>200	>1,000
Vitamin C	30	704

## 3. Experimental

### 3.1. General

Glycitein and daidzein were purchased from Sigma-Aldrich Co. CGTase was purchased from Amano Pharmaceutical Co. Ltd. The NMR spectra were recorded in DMSO-*d*_6_ using a Varian XL-400 spectrometer. The chemical shifts were expressed in *δ *(ppm) units referenced to tetramethylsilane. The HRFABMS spectra were measured using a JEOL MStation JMS-700 spectrometer. HPLC was carried out on a YMC-Pack R&D ODS column (150 × 30 mm) [solvent: CH_3_CN:H_2_O (3:17, v/v); detection: UV (280 nm); flow rate: 1.0 mL/min]. 

### 3.2. Bacterial strain and culture conditions

Culture medium used for growth of *L. delbrueckii* subsp. *bulgaricus* (Okayama University of Science) had the following composition (in grams per liter): 20 g of lactose, 5 g of yeast nitrogen base, 20 g of bacto casitone, 1 g of sorbitan monooleate, 2 g of ammonium citrate, 5 g of sodium acetate, 2 g of K_2_HPO_4_, 0.05 g of MnSO_4_, 0.1 g of MgSO_4_. The cells were grown in the culture medium with continuous shaking on a rotary shaker (120 rpm) at 30 °C.

### 3.3. Production of β-glucosides of glycitein and daidzein by L. delbrueckii

The cultures of *L. delbrueckii* were grown in 500 mL conical flasks containing 200 mL of culture medium at 30 °C. Prior to use for the experiments, the cells were harvested by centrifugation at 8,000 g for 15 min. The β-glucosides of glycitein and daidzein were prepared as follows: substrate (0.2 mmol/flask, 2 mmol total) was added to ten 300 mL conical flasks containing *L. delbrueckii* cells (5 g) and glucose (1 g) in freshly prepared culture medium (100 mL). The mixture was incubated with continuous shaking on a rotary shaker (120 rpm) for 5 days at 30 °C. The reaction mixture was centrifuged at 8,000 g for 15 min to remove the cells and the supernatant was extracted with *n*-butanol. The *n*-butanol fraction was purified by preparative HPLC on YMC-Pack R&D ODS column to give the β-glucoside products.

### 3.4. Production of β-maltooligosides of glycitein and daidzein by CGTase

To a solution containing β-glucosides of glycitein or daidzein (0.1 mmol) and starch (5 g) in sodium phosphate buffer (25 mM, pH 7.0) was added CGTase (100 U). The reaction mixture was stirred at 40 °C for 24 h, and then the mixture was centrifuged at 3,000 g for 10 min. The supernatant was subjected on to a Sephadex G-25 column equilibrated with water to remove CGTase. The fractions containing glycosides were purified by preparative HPLC on YMC-Pack R&D ODS column to give the β-maltooligoside products. Spectral data of new compounds are as follows:

*Glycitein 4'-O-β-maltoside* (**4**): FABMS *m/z*: 631.1640 [M+Na]^+^ (calcd 631.1639 for C_2__8_H_32_O_1__5_Na); ^1^H -NMR (DMSO-*d*_6_): *δ* 3.05-3.80 (12H, m, H-2'', 2''', 3'', 3''', 4'', 4''', 5'', 5''', 6'', 6'''), 3.92 (3H, s, OCH_3_), 5.05 (1H, d, *J *= 3.2 Hz, H-1'''), 5.11 (1H, d, *J *= 7.6 Hz, H-1''), 7.00 (2H, d, *J *= 8.0 Hz, H-3', 5'), 7.46 (1H, s, H-8), 7.51 (2H, d, *J *= 8.0 Hz, H-2', 6'), 7.75 (1H, s, H-5), 8.41 (1H, s, H-2); ^13^C-NMR (DMSO-*d*_6_): *δ* 56.1 (OCH_3_), 60.7 (C-6'''), 60.8 (C-6''), 69.5 (C-4'''), 72.0 (C-2'''), 72.8 (C-5'''), 73.3 (C-2''), 73.5 (C-3'''), 75.3 (C-5''), 75.9 (C-3''), 80.5 (C-4''), 98.0 (C-1''), 100.5 (C-1'''), 101.1 (C-8), 105.0 (C-5), 115.1 (C-3', C-5'), 117.7 (C-10), 122.5 (C-1'), 124.6 (C-3), 130.3 (C-2', C-6'), 148.8 (C-6), 151.0 (C-9), 152.2 (C-7), 153.8 (C-2), 157.3 (C-4'), 178.1 (C-4).

*Glycitein 4'-O-β-maltotrioside* (**5**): FABMS *m/z*: 793.2170 [M+Na]^+^ (calcd 793.2167 for C_34_H_42_O_20_Na); ^1^H-NMR (DMSO-*d*_6_): *δ* 3.09-3.85 (18H, m, H-2'', 2''', 2'''', 3'', 3''', 3'''', 4'', 4''', 4'''', 5'', 5''', 5'''', 6'', 6''', 6''''), 3.92 (3H, s, OCH_3_), 5.05 (1H, d, *J *= 3.2 Hz, H-1''''), 5.07 (1H, d, *J *= 3.0 Hz, H-1'''), 5.12 (1H, d, *J *= 7.6 Hz, H-1''), 7.01 (2H, d, *J *= 8.4 Hz, H-3', 5'), 7.46 (1H, s, H-8), 7.50 (2H, d, *J *= 8.4 Hz, H-2', 6'), 7.76 (1H, s, H-5), 8.41 (1H, s, H-2); ^13^C-NMR (DMSO-*d*_6_): *δ* 56.1 (OCH_3_), 60.5 (C-6''', C-6''''), 60.7 (C-6''), 69.5 (C-4''''), 71.5 (C-5'''), 72.0 (C-2'''), 72.4 (C-2'''', C-5''''), 73.0 (C-3''''), 73.3 (C-3'''), 73.8 (C-2''), 75.1 (C-5''), 75.9 (C-3''), 78.8 (C-4''), 78.9 (C-4'''), 98.1 (C-1''), 101.5 (C-1''', C-1''''), 101.9 (C-8), 105.1 (C-5), 115.0 (C-3', C-5'), 117.6 (C-10), 122.5 (C-1'), 124.5 (C-3), 130.3 (C-2', C-6'), 148.7 (C-6), 151.0 (C-9), 152.2 (C-7), 153.8 (C-2), 157.6 (C-4'), 178.0 (C-4).

*Glycitein 7-O-β-maltoside* (**6**): FABMS *m/z*: 631.1641 [M+Na]^+^ (calcd 631.1639 for C_2__8_H_32_O_1__5_Na); ^1^H-NMR (DMSO-*d*_6_): *δ* 3.09-3.77 (12H, m, H-2'', 2''', 3'', 3''', 4'', 4''', 5'', 5''', 6'', 6'''), 3.90 (3H, s, OCH_3_), 5.07 (1H, d, *J *= 3.2 Hz, H-1'''), 5.19 (1H, d, *J *= 7.6 Hz, H-1''), 7.01 (2H, d, *J *= 8.0 Hz, H-3', 5'), 7.46 (1H, s, H-8), 7.52 (2H, d, *J *= 8.0 Hz, H-2', 6'), 7.76 (1H, s, H-5), 8.41 (1H, s, H-2); ^13^C-NMR (DMSO-*d*_6_): *δ* 56.1 (OCH_3_), 60.2 (C-6'''), 60.9 (C-6''), 69.7 (C-4'''), 72.0 (C-2'''), 72.7 (C-5'''), 73.3 (C-2''), 73.5 (C-3'''), 75.1 (C-5''), 75.9 (C-3''), 79.9 (C-4''), 98.8 (C-1''), 100.6 (C-1'''), 101.0 (C-8), 105.1 (C-5), 115.2 (C-3', C-5'), 117.7 (C-10), 122.5 (C-1'), 124.5 (C-3), 130.3 (C-2', C-6'), 148.8 (C-6), 151.0 (C-9), 152.5 (C-7), 153.8 (C-2), 157.0 (C-4'), 178.0 (C-4).

*Glycitein 7-O-β-maltotrioside* (**7**): FABMS *m/z*: 793.2177 [M+Na]^+^ (calcd 793.2167 for C_34_H_42_O_20_Na); ^1^H-NMR (DMSO-*d*_6_): *δ* 3.05-3.88 (18H, m, H-2'', 2''', 2'''', 3'', 3''', 3'''', 4'', 4''', 4'''', 5'', 5''', 5'''', 6'', 6''', 6''''), 3.92 (3H, s, OCH_3_), 5.07 (1H, d, *J *= 3.2 Hz, H-1''''), 5.09 (1H, d, *J *= 3.2 Hz, H-1'''), 5.19 (1H, d, *J *= 7.6 Hz, H-1''), 7.00 (2H, d, *J *= 8.4 Hz, H-3', 5'), 7.45 (1H, s, H-8), 7.51 (2H, d, *J *= 8.4 Hz, H-2', 6'), 7.76 (1H, s, H-5), 8.41 (1H, s, H-2); ^13^C-NMR (DMSO-*d*_6_): *δ* 56.1 (OCH_3_), 60.5 (C-6''''), 60.7 (C-6'', C-6'''), 69.7 (C-4''''), 71.5 (C-5'''), 72.1 (C-2'''), 72.5 (C-2'''', C-5''''), 73.0 (C-3''''), 73.5 (C-3'''), 73.8 (C-2''), 75.1 (C-5''), 75.7 (C-3''), 79.7 (C-4''), 79.9 (C-4'''), 98.1 (C-1''), 100.5, 100.6 (C-1''', C-1''''), 101.9 (C-8), 105.1 (C-5), 115.0 (C-3', C-5'), 117.6 (C-10), 122.5 (C-1'), 124.5 (C-3), 130.2 (C-2', C-6'), 148.8 (C-6), 151.0 (C-9), 152.5 (C-7), 153.8 (C-2), 157.0 (C-4'), 178.1 (C-4).

### 3.5. Suppressive action on IgE antibody formation

The inhibitory action of β-glycosides of glycitein or daidzein on IgE antibody formation was examined as follows: 7S-globulin was used as the antigen (1 mg/rat), and Al(OH)_3_ and pertussis vaccine were used as the adjuvants (20 mg and 0.6 mL/rat, respectively). Sensitization was made by injection of a mixture (0.6 mL) of the antigen and the adjuvant into the paws of each rat (male, ca. 200 g). Paw edema was measured 24 h after injection and the treated rats were divided in groups with an equal average swelling volume. Each sample was dissolved in physiological saline containing 10% Nikkol and the solution was injected daily into the rat for 11 d starting on the day of grouping. Hydrocortisone was used as the positive control. The amount of IgE was measured by the passive cutaneous anaphylaxis method on the 15th day [17]. The results were expressed as average of plasma IgE level of 7 rats administered a total of 10 mg/kg of each test compound.

### 3.6. DPPH radical scavenging activity

DPPH free-radical scavenging activities of β-glycosides of glycitein or daidzein were determined as follows: DPPH was dissolved in ethanol (500 μM) [21]. The sample solutions were prepared by dissolving each compound in ethanol. To a solution containing various concentrations of each sample (0.1 mL) and ethanol (0.9 mL) was added DPPH solution (1 mL) at room temperature. Vitamin C was used as a positive control. After 20 min at 25 °C, the absorbance was measured at 517 nm. The percentage reduction of the initial DPPH adsorption, *i.e.*, the free-radical scavenging activity, was calculated as follows: E = [(*A*_c _ − *A*_t_)/*A*_c_] × 100, where *A*_t_ and *A*_c_ are the respective absorbance at 517 nm of sample solutions with and without the test compounds. Antioxidant activity was expressed as the 50% inhibitory concentration (IC_50_).

### 3.7. Superoxide-radical scavenging activity

Superoxide was generated by the xanthine-xanthine oxidase system [21]. Reaction mixture contained 4 mM xanthine (50 μL), various concentration of sample in ethanol (50 μL), 2 mM nitro blue tetrazolium (NBT, 50 μL), of 0.3 nkat/mL xanthine oxidase (50 μL) and 0.1 M phosphate buffer (pH 7.4) in a total volume of 2 mL. Vitamin C was used as a positive control. The reaction mixture was incubated at 25 °C for 10 min and the absorbance was read at 560 nm. Percent inhibition was calculated by comparing with control without test compound but containing the same amount of alcohol. The IC_50_ value is shown as the sample concentration at which 50% of superoxide-radical was scavenged.

## 4. Conclusions

The β-maltooligosaccharides of glycitein and daidzein were successfully produced through two-step biocatalytic glycosylation by *L. delbrueckii* and CGTase. The 7-*O*-β-glucoside of glycitein and daidzein and 7-*O*-β-maltoside of glycitein inhibited IgE antibody formation. On the other hand, β-glucosides of glycitein and daidzein exerted DPPH free-radical scavenging activity and supeoxide-radical scaventing activity.
